# LysR-type transcriptional regulator *CARR* represses the expression of *bla*_CAR-2_ and reduces *P. diazotrophicus* resistance to cefalothin, cefuroxime and cefotaxime

**DOI:** 10.3389/fcimb.2025.1616646

**Published:** 2025-09-01

**Authors:** Jiansheng Lin, Chunyan Lin, Jingyang Zheng

**Affiliations:** ^1^ Microbiology Laboratory, Quanzhou Women’s and Children’s Hospital, Quanzhou, China; ^2^ The Affiliated Women’s and Children’s Hospital of Huaqiao University, Quanzhou, China; ^3^ Respiratory Department, Quanzhou Women’s and Children’s Hospital, Quanzhou, China

**Keywords:** *P. diazotrophicus*, LysR-type transcriptional regulator CARR, metallo-β-lactamases (MBLs), *bla*_CAR-2_, antibiotic resistance

## Abstract

**Background:**

*P. diazotrophicus* was isolated from a newborn with D-galactosemia complicated sepsis. A homologous *bla*_CAR-2_ gene, encoding CAR-2, a predicted member of the CAR family subclass B3 metallo-β-lactamases (MBLs), was found in the genome of this strain. This study aimed to explore the identification of a novel CAR-2 protein encoded by the chromosome of *P. diazotrophicus* Pd1 that exhibits the zinc-binding motifs of subclass B3 enzymes and the regulatory pattern of *CARR*, located directly upstream of the *bla*_CAR-2_ gene, on the *bla*_CAR-2_ gene and its impact on antibiotic resistance.

**Methods:**

Antibiotic susceptibility testing was conducted by the plate agar dilution method. Site-directed mutagenesis was conducted using Mut Express II Fast Mutagenesis Kit V2. Kinetic assays were used to determine the hydrolysis of β-Lactam. The construction of bacterial knockout strains was carried out according to the principle of homologous recombination. The detection of the mRNA expression level of the gene was performed by Real-time Quantitative PCR (qPCR).

**Results:**

The minimum inhibitory concentrations (MICs) for *E.coli* DH5α(pHSG398::CAR-2), which expressed *bla*_CAR-2,_ increased significantly for cefalothin, cefuroxime and cefotaxime sodium by 2-, 16- and 32- fold, respectively, which showed that *bla*_CAR-2_ had resistance to these three antibiotics. This protein, which was a MBL, contains two classical zinc-binding sites characteristic of subclass B3, with the amino acid motif His136, His138, His211, Asp140, His141, and His276. Among the six residues, His136, Asp140, and His211 exhibited the highest catalytic activity. We determined that CAR-2 can effectively hydrolyze cefalothin, cefuroxime and cefotaxime. The chloramphenicol resistance of the constructed *E. coli* DH5α strain was significantly reduced in the presence of *CARR* than in the absence of *CARR*. Compared with those for the wild-type *P. diazotrophicus*, MICs of cefalothin, cefuroxime and cefotaxime for the Δ*CARR* strain increased by 8-, 8- and 16-fold, respectively, and the expression level of *bla*_CAR-2_ also increased by approximately 10-fold.

**Conclusion:**

Overall, CAR-2 is a novel subclass B3 MBL of the CAR family that exhibits catalytic activity against cefalothin, cefuroxime and cefotaxime. CARR represses the expression of *bla*_CAR-2_, thereby reducing the resistance of *P. diazotrophicus* to these antibiotics. This provided a theoretical basis for revealing new mechanisms of pathogen resistance.

## Introduction

*Phytobacter diazotrophicus* (*P. diazotrophicus*) belongs to the Enterobacteriaceae family and was initially identified as a gram-negative bacterium that promotes plant growth. Subsequently, this species has been associated with opportunistic infections in humans and nosocomial settings (Pillonetto et al., 2018b; Kubota et al., 2023). The clinical significance of *P. diazotrophicus* has likely been underestimated due to frequent misidentification of clinical isolates as other Enterobacteriaceae (Pillonetto et al., 2018a). β-lactam antibiotics are the primary therapeutic agents for treating infections caused by *P. diazotrophicus*. In the past 2 years, clinical reports have described the emergence of multidrug-resistant *P. diazotrophicus* strains (Hon et al., 2023; Almuzara M et al., 2024). In 2010, The Lancet Infectious Disease published an article on the New Delhi metallo-β-lactamase-1 (NDM-1) superbug, which attracted widespread public and scientific attention (Kumarasamy et al., 2010). The increased global use of antibiotics has contributed to the rise of NDM-1-producing superbugs (Ranjan and Thatikonda, 2021). NDM-1-producing *P. diazotrophicus* strains have been detected in hospitalized patients and healthcare environments (Kubota et al., 2023). Currently, metallo-β-lactamase (MBL)-producing pathogens represent a major threat to clinical treatment options (S et al., 2023).

Clinically, Ambler classifies β-lactamases into four categories: A, B, C, and D (Ambler, 1980). Among them, the active sites of classes A, C, and D are serine residues; they are also known as serine-β-lactamases. In contrast, class B enzymes require metal ion coordination at the active site and are referred to as MBLs. Many novel MBLs have been speculated to originate from environmental bacteria, which serve as a large reservoir of antibiotic resistance genes that can be transferred into pathogenic bacteria (Martínez, 2018). MBLs can hydrolyze a broad spectrum of β-lactam antibiotics, except for monobactams, and their activity can be inhibited by metal ion-chelating agents such as EDTA, phenanthroline, and sulfhydryl compounds (Fisher et al., 2005). Class B MBLs are further divided into three subclasses, B1, B2, and B3, based on their amino acid sequences, structural features, and the number of zinc ion-binding sites (Krco et al., 2023). Subclass B3 is classified separately from B1 and B2 due to its extremely low sequence homology with the other subclasses. Most characterized subclass B3 MBLs have been discovered in environmental bacteria, but have been increasingly identified in clinical samples (Krco et al., 2023). The discovery of Adelaide Imipenemase (AIM-1), a subclass B3 MBL in *Pseudomonas aeruginosa* (*P. aeruginosa*) that efficiently hydrolyzes a wide range of β-lactam antibiotics and performs better than subclass B1 MBLs such as IMP-1 and VIM-2, suggests that the functional significance of subclass B3 MBLs has been underestimated (Yong et al., 2012). Given the increasing prevalence of antibiotic resistance, the absence of effective MBL inhibitors in clinical practice has become an urgent concern (Mojica et al., 2022).

We isolated and cultured gram-negative bacilli from the blood of a newborn with D-galactosemia complicated by sepsis. This strain was successfully identified as *P. diazotrophicus*, named *P. diazotrophicus* Pd1 (Lin et al., 2024). The genome of this strain carries gene_2795, a homolog of *bla*_CAR_. To facilitate its distinction from the previously reported *bla*_CAR-1_ gene (locus number: ECA2849) and its encoded protein CAR-1, our research group named the gene_2795 in *P. diazotrophicus* as *bla*_CAR-2,_ and its encoded protein CAR-2. Previous literature reported that the CAR-1 protein, the CAR family, subclass B3 MBLs, can effectively hydrolyze broad-spectrum β-lactam antibiotics (Stoczko et al., 2008). In the genome of *P. diazotrophicus* Pd1, directly upstream of *bla*_CAR-2_ is gene_2796, which is divergently transcribed relative to *bla*_CAR-2_ gene and encodes a LysR-type transcriptional regulatory protein. This gene was named the CAR-related regulator (*CARR*). However, the mechanism of action of the CAR-2 protein and the CARR regulatory protein in emerging pathogenic bacteria, as well as the regulatory relationship between CARR and the *bla*_CAR-2_ gene, remain unclear.

Herein, we report the identification of a novel CAR-2 protein encoded by the chromosome of *P. diazotrophicus* Pd1 that exhibits the zinc-binding motifs of subclass.

B3 enzymes. By constructing active mutants of the CAR-2 protein, the active site of this protein was determined based on changes in antibiotic susceptibility testing. To clarify the function of the CAR-2 protein in hydrolyzing antibiotics, we conducted kinetic measurements. In this study, through gene knockout and expression analysis, we explored the regulatory pattern of *CARR* on the *bla*_CAR-2_ gene and its impact on antibiotic resistance.

## Materials and methods

### Bacterial strains and culture conditions

*P. diazotrophicus* Pd1, *E. coli* DH5α and *E. coli* BL21(DE3) were preserved in our laboratory. *E. coli* DH5α served as the host for constructing recombinant plasmids. *E. coli* BL21(DE3) was used for the induced expression of the CAR-2 protein. *E. coli* S17-1(λpir) was purchased from Beyotime Biotechnology and was used for constructing suicide plasmid. *P. diazotrophicus* Pd1 was cultured on a blood agar plate at 37°C. The draft complete sequences of *P. diazotrophicus* Pd1 was deposited in NCBI GenBank under the accession number: PRJNA1020661.

### Sequence analysis for CAR-2

To clarify the similarity between predicted CAR-2 and other β-lactamases, we conducted the analysis through the Beta-lactamase database (BLDB)-structure and function BLAST analysis (http://www.bldb.eu:4567/). The signal peptide was predicted through protein signal peptide prediction (https://www.novopro.cn/tools/signalp). The molecular mass and predicted isoelectric point of CAR-2 were estimated using ExPASy(https://web.expasy.org/compute_pi/).

### Expression of *bla*_CAR-2_ in *E. coli* DH5α

A pHSG398::CAR-2 construct was generated to test the β-lactam substrate specificity of CAR-2, as described previously (Ranjan et al., 2021). High-fidelity PCR was used to amplify the complete coding frame sequence of *bla_CAR-2_* with the forward primer EcoRI-*bla*_CAR-2_-F and the reverse primer PstI-*bla*_CAR-2_-R. The primer design was carried out using Primer 5 software with the following parameter settings: annealing temperature is 58–62°C, primer length is 18–24 bp, and GC content is 40–60%. Primer sequences are shown in **Supplementary Table S1**. High-fidelity PCR was purchased from Vazyme. The total reaction volume of 50 µL consisted of 20 µL ddH_2_O, 25 µL 2× Phanta Max Master Mix, 2 µL primer F, 2 µL primer R, and 1 µL template DNA. The thermal cycle was programmed for 3 min at 95°C for pre-denaturation, followed by 32 cycles of 15 s at 95°C for denaturation, 15 s at 60°C for annealing, 60 s at 72°C for extension; a final extension was conducted for 5 min at 72°C. The PCR product and the pHSG398 plasmid (Takara) were double-digested with EcoRI and PstI, and then the PCR product was ligated to the pHSG398 plasmid using T4 ligase. The restriction enzymes EcoRI and PstI were purchased from Thermo Fisher Scientific. Using the forward primer EcoRI-*bla*_CAR-2_-F and the reverse primer PstI-*bla*_CAR-2_-R, PCR was performed to verify that the recombinant pHSG398 plasmid was successfully connected and transformed into *E. coli* DH5α, which was named *E.coli* DH5α(pHSG398::CAR-2). This recombinant strain expresses *bla*_CAR-2_ under the control of the lac promoter. Similarly, using the forward primer Test-pHSG398-F and the rear primer Test-pHSG398-R, PCR was performed to confirm the successful transformation of the empty pHSG398 plasmid into *E. coli* DH5α, which was named *E.coli* DH5α(pHSG398). This strain was used as a negative control.

### Antibiotic susceptibility testing

The minimum inhibitory concentration (MIC) values of susceptibility of strains to 14 kinds of β-lactam antibiotics including ampicillin, piperacillin, carbenicillin, cefalotin, cefoxitin, cefuroxime, ceftazidime, cefotaxime, ceftazidime/clavulanic acid, cefepime, aztreonam, imipenem, meropenem, and ertapenem were detected by the Mueller-Hinton (MH) agar plate dilution method. Antibiotics were purchased from Aladdin Biochemical Technology Co., Ltd. In brief, we first revived strains on a blood plate for 24 hours, then picked single clones and subcultured them for 24 hours. Subsequently we added each antibiotic with a serial dilution concentration to an empty plate, and then poured 25 mL of MH agar medium at about 50°C into each plate. Next, we adjusted the bacterial suspension concentration to 0.5 McFarland turbidity standard with a McFarland turbidimeter, and titrated 1 μL of the bacterial suspension onto the surface of the MH agarplates. After air-drying, finally we placed the plates in an incubator at 37°C, and observed the MIC values of each strain 20 hours later. *E. coli* ATCC8739 was selected as the quality control bacterium. Repeat the biological experiment three times.

For determination of *bla*_CAR-2_ MBL activity, MIC values of 14 kinds of β-lactam antibiotics were also determined in the presence of 0.2 mM EDTA to demonstrate the reduction in the *bla_CAR-2_* activity due to metal chelation.

### Construction of mutations at the key active sites of the CAR-2 protein

To test the key active sites of the CAR-2 protein, we constructed six single mutants (H136A, H138A, H211A, D140A, H141A, and H276A) in *E. coli* DH5α. Site-directed mutagenesis was conducted using Mut Express II Fast Mutagenesis Kit V2 (Vazyme Biotech Co., Ltd). Briefly, the codons corresponding to the amino acid residues His136, His138, His211, Asp140, His141, and His276 were replaced with the codon GCC, corresponding to alanine(A), at the 5’ end of the primers. Primer sequences are listed in **Supplementary Table S1**. Using pHSG398::CAR-2 plasmid DNA as a template, six single mutant plasmids—pHSG398::CAR-2(H136A), pHSG398::CAR-2(H138A), pHSG398::CAR-2(H211A), pHSG398::CAR-2(D140A), pHSG398::CAR-2(H141A), and pHSG398::CAR-2(H276A)—were amplified with the primer pairs mentioned above. Subsequently, these plasmids were individually transformed into *E. coli* DH5α. Sanger sequencing (first-generation sequencing) using the forward primer Sanger-F and the rear primer Sanger-R was performed to determine the ~650 bp region containing the mutation site. Sequence alignment using the Chromas software confirmed the successful construction of all six single mutant strains.

### Induced expression and purification of recombinant CAR-2 protein

Firstly, we used high-fidelity PCR to amplify the coding frame sequence (removing the signal peptide sequence) of *bla*_CAR-2_ with the forward primer NdeI-CAR-2-F and the reverse primer XhoI-CAR-2-R. The restriction enzymes NdeI and XhoI were purchased from Thermo Fisher Scientific. Primer sequences was showed in **Supplementary Table S1**. The PCR product and pET-21b(+) were double-digested with NdeI and XhoI, and then PCR product ligated to the pET-21b(+) plasmid using T4 ligase. Using the forward primer NdeI-CAR-2-F and the reverse primer XhoI-CAR-2-R, PCR was performed to verify that the Recombinant pET-21b(+) plasmid was successfully transformed into *E. coli* BL21(DE3), which was named *E. coli* BL21(DE3) (pET-21b-CAR-2). A single clone of *E. coli* BL21(DE3) (pET-21b-CAR-2) was added to 500 mL of liquid LB medium and cultured at 37°C and 220 rpm/min for 9 hours. Subsequently, we added IPTG to the bacterial culture to a final concentration of 0.5 mM and then incubated it on a shaker overnight at 37°C.The bacterial culture was collected by centrifugation at 5000rpm/min for 5 minutes and resuspended in a Tris·HCl buffer solution containing 20 mM imidazole. The bacteria were lysed by sonication (power 30%, sonication time 45 min, on for 3 s and off for 3 s).After centrifuging the lysate at 12,000 rpm/min for 20 minutes, it was filtered through a sterilizing filter. In the affinity chromatography column, the filtrate was mixed with Ni^2+^-NTA Agarose 6FF (Shanghai Yuanye Bio - Technology Co., Ltd) for 30 minutes. A Tris·HCl buffer solution containing 20 mM imidazole was used as the washing buffer to elute the non-target proteins. A Tris·HCl buffer solution containing 50 mM, 100 mM, 200 mM, 300 mM or 400 mM imidazole was used as the elution buffer to elute the target protein bound to the nickel column. The purification effect was analyzed by SDS-PAGE. The His-tagged CAR-2 protein was dialyzed overnight in a PBS buffer solution (pH 7.4) using a dialysis bag. The concentration of the recombinant CAR-2 protein was determined by a Nano-300 Micro-Spectrophotometer.

### Determination of kinetic parameters

Kinetic assays were conducted with a UV-1100 spectrophotometer as previously described (Stoczko et al., 2008).β-Lactam hydrolysis was detected by monitoring the variation in absorbance using the characteristic molecular extinction coefficient to substrates: cefalothin (Δϵ_260nm_=-14,300 M^-1^cm^-1^); cefuroxime (Δϵ_274nm_=-18,600 M^-1^cm^-1^); cefotaxime sodium (Δϵ_264nm_=-11,625 M^-1^cm^-1)^. The assays were conducted at 25°C with 3 ml of reaction mixture in 50mM HEPES buffer, pH 7.5 containing CAR-2 protein (0.634µM for cefalothin;1.27µM for cefuroxime; 0.634µM for cefotaxime sodium), 50µM ZnSO_4_.The steady-state kinetic parameters (*v*_max_ and *k*_m_) were calculated after direct fit of the Michaelis-Menten equation on the experimental data with the program GraphPad. The value of *k*_cat​_ was obtained by dividing *v*_max_ by the CAR-2 protein concentration.

### Construction of *E.coli* DH5α (pBluescript/IR111-Cm^R^) and *E.coli* DH5α (pBluescript/CARR-IR111-Cm^R^) for MIC experiments

To demonstrate that CARR binds IR111 (the 111- bp sequence of the interval between *CARR* and *bla*_CAR-2_) and inhibits the expression of the chloramphenicol resistance gene *Cm* ^R^, we constructed *E.coli* DH5α (pBluescript/IR111-Cm^R^) and *E.coli* DH5α (pBluescript/CARR-IR111-Cm^R^). In brief, the IR111 fragment and IR111-*CARR* fragment were amplified from *P*. *diazotrophicus* Pd1 genomic DNA using primers IR111-F, IR111-R and IR111-F, IR111-*CARR*-R, respectively. The *Cm* ^R^ coding frame sequence was amplified from the pDM4 plasmid using primers Cm^R^-F and Cm^R^-R. IR111 and Cm^R^ were ligated to pBluescript double-digested with KpnI and SacII by ligase independent cloning (LIC) with ClonExpress MultiS One Step Cloning Kit (Vazyme Biotech Co., Ltd).Likewise, IR111-*CARR* and Cm^R^ were ligated into the pBluescript vector. The two recombinant plasmids were subsequently introduced into *E.coli* DH5α by heat shock. Finally, the MIC values of chloramphenicol in *E. coli* DH5α (pBluescript/IR111-Cm^R^) and *E. coli* DH5α (pBluescript/CARR-IR111-Cm^R^) were determined by the plate agar dilution method.

### Construction of the *CARR* deletion mutant strain(Δ*CARR*)

The construction of Δ*CARR* was carried out according to homologous recombination. In brief, a upstream 583 bp fragment and a downstream 635 bp fragment of *CARR* were amplified from *P*. *diazotrophicus* Pd1 genomic DNA with primers *CARR*-up-F, *CARR*-up-R, and primers *CARR*-down-F, *CARR*-down-R, respectively. These two fragments and a tetracycline resistance cassette, which was amplified with *CARR*-TC-F and *CARR*-TC-R, were ligated into Suicide pDM4 plasmid by LIC with ClonExpress MultiS One Step Cloning Kit. The recombinant plasmids were subsequently introduced into *P*. *diazotrophicus* Pd1 strains by electrotransformation. The bacterial solution was subsequently spread on LB agar plates containing tetracycline (20µg/mL) and piperacillin (24µg/mL). A single colony was subcultured for 5 generations in liquid LB medium. Then, 20µl of bacterial culture was spread on LB agar plates containing tetracycline (20µg/mL). Chromosomal DNA of the transformants was checked by PCR with primers *CARR*-Delete-F and *CARR*-Delete-R. The primers used in this study are listed in **Supplementary Table S1**.

### RNA isolation and quantitative PCR

RNA extraction was performed using the FreeZol Reagent (Vazyme Biotech Co., Ltd) according to the Manufacturer’ instructions. The RNA concentration was analyzed using a Nano-300Micro-Spectrophotometer. Subsequently, 500 ng of total RNA was reverse transcribed into cDNA with the HiScript III RT SuperMix for qPCR(+gDNA wiper) kit (Vazyme Biotech Co., Ltd). Real time quantitative PCR (qPCR) was performed using a fully-automated real-time fluorescent quantitative PCR analysis system Gentier 96 (Tianlong Technology Co., Ltd, Xi’an, China) with ChamQ Blue Universal SYBR qPCR Master Mix (Vazyme Biotech Co., Ltd) in 50 ng of cDNA. The primers used are detailed in **Supplementary Table S1**. Data normalization was performed using 16S rRNA (a housekeeping gene), and relative gene expression was calculated using the 2^–ΔΔCt^ approach.

### Statistical analysis

Student’s t-tests was used to evaluate the statistical significance of differences. A *P*-value < 0.05 was considered to indicate a statistically significant difference.

## Results

### Sequence features of CAR-2

**Figure 1:**
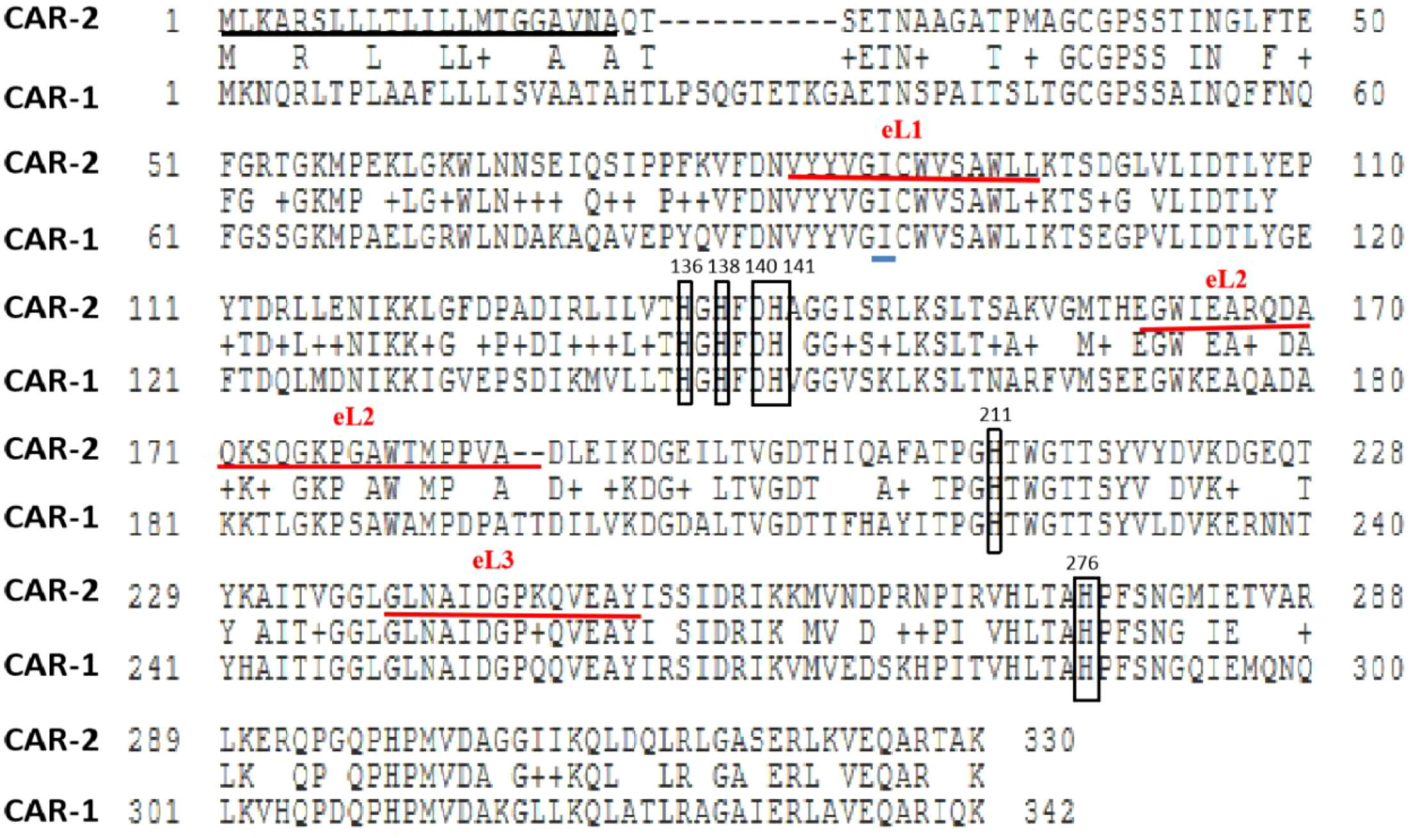
Sequence alignment analysis of the amino acid sequences between CAR-2 and CAR-1 note: Score 433.72, E value 5.36×10^-154^, Identities 208/342 (60.82),Gaps 12/342 (3.51),Positives 256/342 (74.85).The square represents the zinc-binding sites. The signal peptide was marked with a black underline. External loops (eLs) were marked with a red underline. The light blue underlining indicated the position of Thr57Ile as reported in the literature (Lin et al., 2024).

The 990-bp *bla*_CAR-2_ encodes a 330-residue protein, which the highest sequence identity (60.82%) with subclass B3 MBLs CAR-1, as shown in **Figure 1**. Compared with that in CAR-1, the N-terminus of CAR-2 lacks a segment of 10 residues, LPSQGTETKG. CAR-2 shows lower identity to other subclass B3 MBLs, with identity scores ranging from 27.99% (AIM-1) to 34.85% (BJP-1). The novel CAR-2 was predicted to have two zinc-binding motifs: His136, His138, His211, Asp140,His141, and His276, as shown in **Figure 1**. The estimated theoretical isoelectric point and molecular weight of CAR-2 without the predicted signal peptide were 7.28 and 33.717 kDa, respectively. CAR-2 is speculated to possess three external loops (eLs), namely eL1- eL3 (Stoczko et al., 2008; Yun et al., 2022), as shown in **Figure 1**.

### Characterization of the MBL CAR-2

To clarify the substrate specificity of CAR-2, we constructed *E.coli* DH5α(pHSG398::CAR-2), which expressed CAR-2, and tested the MIC values for 14 β-lactam antibiotics. Compared with those for *E.coli* DH5α and *E. coli* DH5α(pHSG398), the MIC values for *E.coli* DH5α(pHSG398::CAR-2) increased significantly only for cefalothin, cefuroxime and cefotaxime sodium by 2-, 16- and 32- fold, respectively (**Table 1**). This result indicates that cefalothin, cefuroxime and cefotaxime are substrates of CAR-2. To confirm that CAR-2 is a MBL, we measured MICs in the presence of EDTA. Compared with those for *E. coli* DH5α(pHSG398::CAR-2) without EDTA, the MIC values for cefalothin, cefuroxime and ceftriaxone sodium in the presence of EDTA dropped to baseline levels (**Table 1**). This confirms that EDTA inhibited CAR-2 catalytic activity, indicating that CAR-2 is a MBL.

**Table 1:** The MIC values of antibiotics for different transformants of *E. coli* DH5α.

Antibiotic	MIC (µg/mL)			
	*E.coli* DH5α	*E.coli* DH5α (pHSG398)	*E.coli* DH5α (pHSG398::CAR-2)	*E.coli* DH5α (pHSG398::CAR-2)+EDTA^*^
Ampicillin	16	16	16	16
Piperacillin	0.5	0.5	0.5	0.5
Carbenicillin	4	4	4	4
Cefalothin	4	4	8	4
Cefoxitin	8	8	8	8
Cefuroxime	2	2	32	2
Cefotaxime sodium	0.0156	0.0156	0.5	0.0156
Ceftazidime	0.125	0.125	0.125	0.125
Ceftazidime/ Clavulanic Acid	0.125	0.125	0.125	0.125
Cefepime	0.32	0.32	0.32	0.32
Aztreonam	0.032	0.032	0.032	0.032
Meropenem	0.125	0.125	0.125	0.125
Ertapenem	0.032	0.032	0.032	0.032
Imipenem	0.25	0.25	0.25	0.25

*Indicates that the final concentration of EDTA is 200 µM.

To determine the catalytic active site of CAR-2, we constructed six single mutants: *E.coli* DH5α[pHSG398::CAR-2(H136A)], *E.coli* DH5α[pHSG398::CAR-2(H138A)], *E. coli* DH5α [pHSG398::CAR-2(H211A)], *E. coli* DH5α [pHSG398::CAR-2(D140A)], *E. coli* DH5α[pHSG398::CAR-2(H141A)], and *E. coli* DH5α[pHSG398::CAR-2(H276A)], as shown in **Supplementary Figure S1**. Subsequently, we tested the MICs for cefuroxime and cefotaxime sodium. Cefuroxime susceptibility results revealed that compared with *E. coli* DH5α(pHSG398::CAR-2), mutants H136A, D140A and H211A showed the greatest MIC reduction- dropping the baseline levels, H138A and H276A showed moderate decreases, and H141A the least (**Table 2**). Cefotaxime MICs mirrored this trend. We conclude that these six residues are active sites, with H136, D140, and H211 being the primary zinc-binding sites.

**Table 2:** The MIC values of antibiotics for various mutants of CAR-2 on the pHSG398 plasmid in *E. coli* DH5α.

Various mutants strains	MIC (µg/mL)	
	Cefuroxime	Cefotaxime sodium
H136A	2	0.016
H138A	4	0.032
D140A	2	0.016
H141A	8	0.064
H211A	2	0.016
H276A	4	0.032

### CAR-2 hydrolyzed cefalothin, cefuroxime and cefotaxime

We successfully induced the recombinant CAR-2 protein, and the size of this protein is consistent with the expected molecular weight of approximately 35 kDa, as shown in **Supplementary Figure S2**. To demonstrate that CAR-2 acted as a MBL can hydrolyze cefalothin, cefuroxime and cefotaxime, we performed CAR-2 enzymatic kinetic experiments. *k*_cat_ was 0.84S^-1^ and *k*_cat_/*k*_m_ was 1.32×10^4^ M^-1·^S^-1^ for cefalothin; *k*_cat_ was 0.21 S^-1^ and *k*_cat_/*k*_m_ was 4.26×10^3^ M^-1·^S^-1^ for cefuroxime; and *k*_cat_ was 0.61 S^-1^, *k*_cat_/*k*_m_ was 1.87×10^4^ M^-1·^S^-1^ for cefotaxime sodium, as shown in **Table 3**. When 1 mM of EDTA was added to the above reaction solution, no activity was observed, indicating that the CAR-2 enzyme requires zinc ions.

**Table 3:** Kinetic parameters of CAR-2.

Substrate	*V*_max_(µM^·^S^-1^)	*k*_cat_ (S^-1^)	*k*_m_(µM)	*k*_cat_/*k*_m_(M^-1·^S^-1^)
Cefalothin	0.53	0.84	63.78	1.32×10^4^
Cefuroxime	0.27	0.21	49.28	4.26×10^3^
Cefotaxime sodium	0.39	0.62	33.08	1.87×10^4^
Cefepime	ND	NH	ND	ND

NH, no hydrolysis detected; ND, data could not be determined.

### *CARR* inhibited the expression of *bla*_CAR-2_

**Figure 2:**
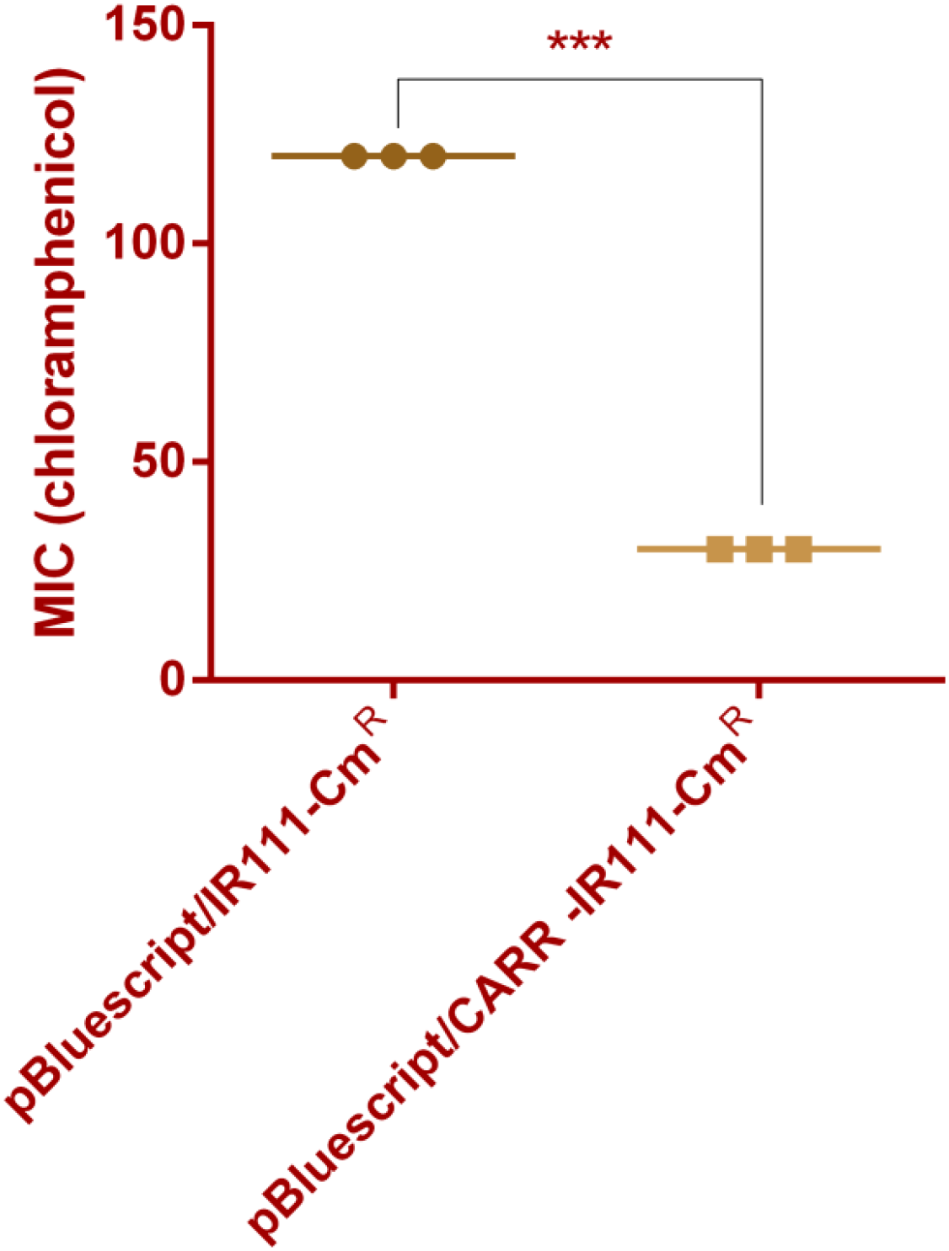
MIC values of *E.coli* DH5α (pBluescript/IR111-Cm^R^) and *E.coli* DH5α (pBluescript/CARR-IR111-Cm^R^) for chloramphenicol (*** denotes *P* < 0.001).

**Figure 3:**
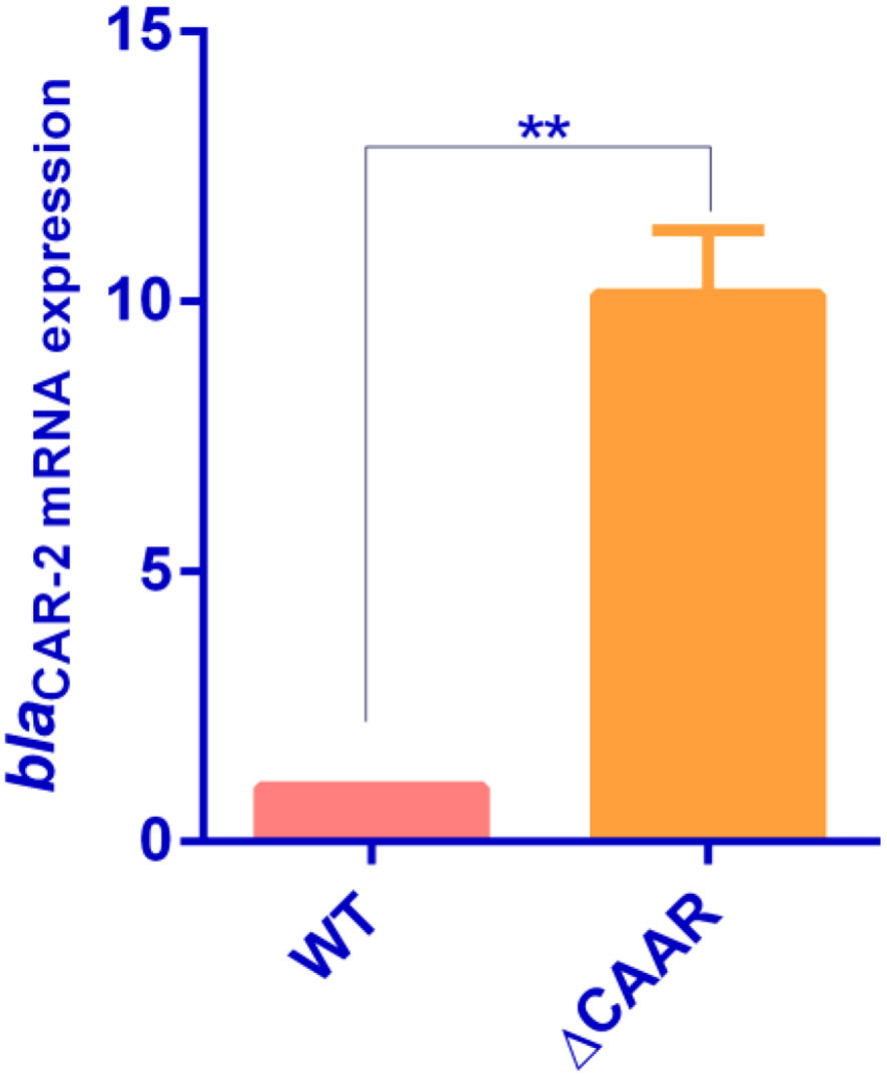
Determination of mRNA expression of *bla_CAR-2_* in *P. diazotrophicus* Pd1 wild-type strain and Δ*CARR* (** denotes *P* < 0.01).

Through alignment analysis using DNAMAN software, the amino acid sequence of this CARR showed 63.76% similarity to that of the LysR-type transcriptional regulator (ECA2848)) located directly upstream of CAR-1. However, the CARR protein was poorly conserved with β-lactamase transcriptional regulators such as AmpR, VarR, and NmcR. The antibiotic sensitivity results showed that the MIC values ​of chloramphenicol for *E.coli* DH5α(pBluescript/IR111-Cm^R^) and *E.coli* DH5α(pBluescript/CARR-IR111-Cm^R^) were 120 µg/mL and 30 µg/mL, respectively, as shown in **Figure 2**. In the presence of *CARR*, the MIC of chloramphenicol decreased 4-fold (*P* < 0.001). These results indicate that *CARR* likely inhibits *bla*_CAR-2_ expression through the IR111 promoter. We successfully constructed the *CARR* deletion mutant strain, as shown in **Supplementary Figure S3**. To further prove that *CARR* inhibits the expression of *bla*_CAR-2_, we used qPCR to detect the mRNA expression of 16S rRNA and *bla*_CAR-2_ in *P. diazotrophicus* Pd1 wild-type strain andΔ*CARR*, as shown in **Supplementary Figure S4**. Compared with that of the wild-type strain, the mRNA expression of *bla*_CAR-2_ in the Δ*CARR* strain increased approximately 10-fold (*P* < 0.01), as shown in **Figure 3**, indicating that *CARR* inhibited *bla*_CAR-2_ expression.

### *CARR* reduced resistance to cefalothin, cefuroxime and cefotaxime

In order to clarify the association between *CARR* and cefalothin, cefuroxime and cefotaxime sodium, the MIC values of cefalothin, cefuroxime and cefotaxime sodium of *P. diazotrophicus* wild-type strain and Δ*CARR* strain were determined. Compared with those of the wild-type strain, the MIC values of the Δ*CARR* strain for cefalothin, cefuroxime and cefotaxime sodium increased 8-,8- and 16-fold, respectively, as shown in **Table 4**. These results demonstrated that *CARR* reduced the resistance to cefalothin, cefuroxime and cefotaxime sodium. Therefore, we conclude that *CARR* inhibits the expression of *bla*_CAR-2_, which reduces the resistance of *P. diazotrophicus* to cefalothin, cefuroxime and cefotaxime.

**Table 4:** Analysis of the impact of knocking out *CARR* on the MIC values of cefalothin, cefuroxime and cefotaxime in *P. diazotrophicus*.

Various strains	MIC (µg/mL)		
	Cefalothin	Cefuroxime	Cefotaxime sodium
WT	4	4	0.0156
Δ*CARR*	32	32	0.25

## Discussion

Subclass B1 prefers penicillin and cephalosporins as substrates, subclass B2 prefers carbapenems as substrates, and subclass B3 prefers penicillin as a substrate (Bush and Jacoby, 2010). The best substrates for CAR-1 were cephalothin, cefuroxime, and cefotaxime (Stoczko et al., 2008). The substrates for CAR-2 were also cephalothin, cefuroxime and cefotaxime. The CAR family MBLs prefer a small number of cephalosporins as substrates. AIM-1, as another member of subclass B3 MBLs, efficiently catalyzes carbapenemases. Therefore, evidently, the substrates of subclass B3 MBLs are quite different.

Subclasses B1 and B3 bind to two zinc ions, whereas subclass B2 binds to a single zinc ion (Bebrone, 2007). For subclasses B1 and B3, the first zinc ion coordinates and binds with the residues His116, His118, and His121; the second zinc ion coordinates and binds with the residues Asp120, Cys221, and His263 for subclass B1. For subclass B3, the second zinc ion coordinates and binds with the residues Asp120, His121, and His263 (Bebrone, 2007; Somboro et al., 2018). However, unlike that in subclasses B1 and B2, considerable diversity exists within the active site motif of subclass B3, such as the non-classical binding sites H116Q and H116E in the first zinc ion and a new combination (H118R, H121Q, and H263K) in the second zinc ion (Berglund et al., 2021). Different from the direct enzymatic activity analysis of protein point mutations (Lee et al., 2019), we directly used the constructed point mutation strains to judge the enzymatic activity by observing drug sensitivity changes, which omitted the protein inducible expression process. Unlike the active-site mutants of PNGM-1 (a subclass B3 MBL), which completely lack catalytic activity (Lee et al., 2019), the H138A and H141A mutants of the CAR-2 protein exhibit partial activity, indicating the uniqueness of the working mode of the active site in the CAR-2 protein. This study determined that CAR-2 has two classical zinc-binding motifs, His136, His138, His211, Asp140, His141, and His276.

The monometallic (zinc) form of subclass B2 MBLs exhibits a high degree of specificity for carbapenem antibiotics (Garau et al., 2005; Fonseca et al., 2011). Similar to AIM-1, GOB-1 belonging to subclass B3 MBLs is also effective against a broad range of β-lactam antibiotics, in particular carbapenems. Thus, it is possible that GOB-1 MBL is active in both mono- and bi-metallic form states (Morán-Barrio et al., 2007; Lisa et al., 2010), which contributes that the substrate spectrum of GOB-1 covers a broad range of β-lactam antibiotics. Based on the fact that the antibiotic susceptibility profiles of CAR-1 and CAR-2 showed no significant correlation with carbapenem resistance, we inferred that CAR family subclass B3 MBLs occurred in bi-metallic form state, which could contribute that the substrates of CAR family subclass B3 MBLs primarily cover cephalosporin antibiotics.

The substrate-binding pocket differs among the three subclasses and is especially important for substrate specificity and drug resistance (Yun et al., 2022). In the superimposed crystal structures of MBLs, there were conserved secondary structures of 5 α-helices and 13 β-strands as the core scaffold. The four loops of L1–4 coordinate the catalytic zinc ion(s), and the three eL1–3 formed a substrate-binding pocket. According to the report of CAR-1, the substitution of the conserved site from Thr57 to Ile in subclass B3 MBLs was considered not to affect the function (Stoczko et al., 2008). However, we believed that the substitution at this site was highly likely to affect the substrate specificity of proteins in the CAR family, because this site was located in the eL1 regions of both CAR-1 and CAR-2, as shown in **Figure 1**.The long N-terminal tail forming eL1 showed varied relative positions in the different B3 MBL structures of L1, GOB-1, CSR-1, and AIM-1,which could affect the catalytic activity (Pedroso et al., 2020). Compared with the high catalytic activities of CAR-1 to cefalothin, cefuroxime and cefotaxime (with *K*_cat_/*K*_m_ values of 1.2×10^6^ M^-1^·S^-1^, 3.6×10^6^ M^-1^·S^-1^ and 1.5×10^6^ M^-1^·S^-1^ respectively), the catalytic activity of CAR-2 is significantly decreased. There are two reasons for this difference: firstly, the signal peptide of the recombinant CAR-2 has been removed; secondly, CAR-2 lacks 10 amino acid residues at the N-terminus, which both affected the formation of the N-terminal loop at the N-terminus and the catalytic activity. Importantly, the amino acid residues of the eL2 loop in CAR-2 differed significantly from those in CAR-1, as shown in **Figure 1**, which may have affected the substrate specificity of CAR-2.

The fifth gene, upstream of *bla*_CAR-2_ gene, encodes an IS3 family transposase, IS Ehe3, which can promote the horizontal movement of *bla*_CAR-2_ between the genomes of different bacteria. This result was consistent with previous findings stating that *bla*_CAR-1_ can be horizontally transmitted between bacteria via a horizontally acquired genomic island (HAI12) (Stoczko et al., 2008). NCBI database analysis revealed that the coexistence of *bla*_CAR-2_ and *CARR* was found only in certain strains of *P. diazotrophicus* and *Phytobacter ursingii* (*P. ursingii*) within the genus *Phytobacter*. However, the coexistence pattern of the homologues of *bla*_CAR-2_ and *CARR* is common in the genus *Pectobacterium*. Therefore, the coexistence of *bla*_CAR-2_ and *CARR* in the genus *Phytobacter* could have evolved from *Pectobacterium*. According to the reports in the literature on the method of gene knockout in *P. diazotrophicus (* Wang et al., 2019), we successfully constructed the *CARR* gene knockout strain. This study is the first to explore the role of the *CARR* gene in drug resistance mechanisms. This study further confirmed that the expression of *bla*_CAR-2_ was inhibited by *CARR* in *P. diazotrophicus*. The limitation of this study lies in the failure to further clarify whether the homologues of *CARR* can inhibit the expression of the homologues of *bla*_CAR-2_ in the strains mentioned above. The metabolic trade-off theory is an important concept in the fields of ecology, evolutionary biology (Miller and O’Dwyer, 2024; Wang et al., 2024). Its core idea is that the energy and resources of organisms are limited. When these resources are allocated to one life activity (such as growth, reproduction, defense, etc.), the investment in other life activities will inevitably be reduced, thereby forming a “trade-off relationship” where one increases and the other decreases. *CARR* may repress antibiotic resistance to prioritize nitrogen fixation in *P. diazotrophicus*. This aligns with metabolic trade-off theory, where resistance genes impose fitness costs (Rajer and Sandegren, 2022).

AmpC synthesis is activated in the presence of β-lactam antibiotics as a result of AmpR derepression. This mechanism involves disruption of the peptidoglycan by β-lactams that leads to increased periplasmic accumulation of cell wall precursors and degradation products (muropeptides). Within the cytoplasm, muropeptides derepress AmpR leading to induction of AmpC (Dietz et al., 1997; Jacobs et al., 1997). The inhibition of the metallo-β-lactamase VarG by the LysR type transcription factor VarR can be relieved in the presence of penicillin G (Lin et al., 2017). However, in the presence of piperacillin (0.25µg/mL) or ampicillin (6µg/mL), the MIC values of chloramphenicol for *E. coli* DH5α (pBluescript/IR111-Cm^R^) and *E.coli* DH5α (pBluescript/CARR-IR111-Cm^R^) remained unchanged compared with those in the absence of β- lactam antibiotics. This indicates that muropeptides cannot induce the expression of *bla*_CAR-2_ by inhibiting *CARR*. In comparison to AmpR/AmpC, penicillin antibiotics fail to relieve CARR-mediated repression of *bla*_CAR-2_.The inducible expression of *bla*_CAR-2_ requires further in-depth research.

## Conclusion

CAR-2 is a novel CAR family MBL that exhibits catalytic activity against cephalothin, cfuroxime and cefotaxime, and is strongly inhibited by EDTA. These six residues His136, His138, His211, Asp140, His141, and His276 are active sites, with His136, Asp140, and His211 being the primary zinc-binding sites. The inhibition of *bla*_CAR-2_ expression by CARR may act directly through the *bla*_CAR-2_ promoter, which reduces the resistance of *P. diazotrophicus* to cephalothin, cefuroxime and cefotaxime. This provided a theoretical basis for revealing new mechanisms of pathogen resistance.

## Data Availability

The draft complete sequences of *P. diazotrophicus* Pd1 was deposited in NCBI GenBank under the accession number: PRJNA1020661.
