# Supplementary material for: LysR-type transcriptional regulator CARR represses the expression of bla CAR-2 and reduces P. diazotrophicus resistance to cefalothin, cefuroxime and cefotaxime
Source: Front Cell Infect Microbiol. 2025 Sep 1;15:1616646. doi: 10.3389/fcimb.2025.1616646 (PMC12434095; doi:10.3389/fcimb.2025.1616646)
Supplement: Supplementary file 1 [file Table1.docx]

**Table S1 Primer names and sequences in this subject**

| **Primer name** | **Primer sequence(5’-3’)** |
| --- | --- |
| EcoRI-*bla*_CAR-2_-F | GGGAATTCATGCTGAAAGCCCGCTCTCTT |
| PstI-*bla*_CAR-2_-R | AACTGCAGCTACTTCGCCGTCCTGGCCTGCT |
| Test-pHSG398-F | CATTAATGCAGCTGGCACGA |
| Test-pHSG398-R | AAGGCGATTAAGTTGGGTAACG |
| H136A-F | GTAACGgccGGTCATTTTGATCACGCTGGA |
| H136A-R | AAATGACCggcCGTTACCAGAATCAATCTGATATCAGC |
| H138A-F | GCATGGTgccTTTGATCACGCTGGAGGCA |
| H138A-R | GATCAAAggcACCATGCGTTACCAGAATCAATC |
| D140A-F | TCATTTTgccCACGCTGGAGGCATTTCTCG |
| D140A-R | CAGCGTGggcAAAATGACCATGCGTTACCAGAA |
| H141A-F | TTTTGATgccGCTGGAGGCATTTCTCGCC |
| H141A-R | CTCCAGCggcATCAAAATGACCATGCGTTACCA |
| H211A-F | AACACCTGGAgccACCTGGGGCACTACATCATATGT |
| H211A-R | AGGTggcTCCAGGTGTTGCAAACGCC |
| H276A-F | CAgccCCTTTCAGCAATGGAATGATTGA |
| H276A-R | ATTGCTGAAAGGggcTGCAGTGAGGTGTACTCTTATCGG |
| Sanger-F  Sanger-R | GAGACGAAGCTGGTCTAGTTGCT  AAGTGACGGCCTGGTTTTAATC |
| NdeI-CAR-2-F | GGAATTCCATATGGCTCAGACATCAGAAACCAACG |
| XhoI-CAR-2-R | CCGCTCGAGCTTCGCCGTCCTGGCCT |
| Cm^R^-F | ctatagggcgaattgggtaccATGATCATATCGTCAATTATTACCTCCA |
| Cm^R^-R | caatggaatgcaacATGGAGAAAAAAATCACTGGATATACC |
| IR111-F | ctccatGTTGCATTCCATTGTAGATTGAACA |
| IR111-CARR-R | aaagctggagctccaccgcggCTAAGAACGGATGATATCGGTTAACA |
| IR111-R | aaagctggagctccaccgcggATTTCCCCTAACGTCCGTTTTG |
| *CARR*-up-F | tgtggaatcccgggagagctcTGATCAAAATGACCATGCGTTAC |
| *CARR*-up-R | acgacaagcttGGCTGTAAGACGGTCCATATTTCC |
| *CARR*-TC-F | cttacagccAAGCTTGTCGTGTTAAAGACCTCC |
| *CARR*-TC-R | tatcggtAAGCTTTTGGCGGGTGTCG |
| *CARR*-down-F | ccgccaaaagcttACCGATATCATCCGTTCTTAGGG |
| *CARR*-down-R | cactagtggggcccttctagaACCGAAACATCCGGCTGAG |
| *CARR*-Delete-F | TTTTCATCAATGTGGTCGAACTG |
| *CARR*-Delete-R | GCGGGTCAGCAAACGTGA |
| 16S rRNA-F | CAGCCATGCCGCGTGTA |
| 16S rRNA-R | TAATTCCGATTAACGCTTGCAC |
| q*bla*_CAR-2_-F | AGACGAAGCTGGTCTAGTTGCTT |
| q*bla*_CAR-2_-R | ACATCAGCAG TATTGATCGCATC |

Notes: The restriction enzyme recognition sites are underlined. Lowercase letters indicate overlapping bridging sequences.


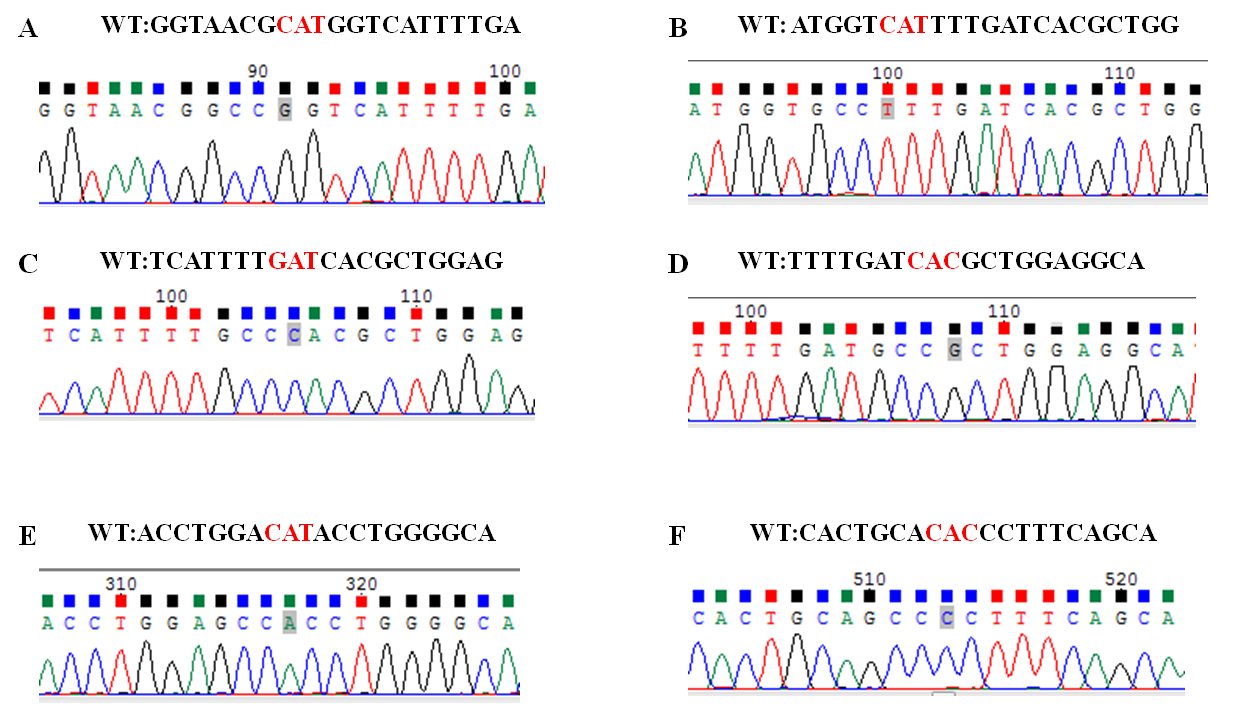


**Figure S1 The sequencing validation maps for the successful construction of six active-site point mutations**  A represents H136A, B represents H138A, C represents D140A, D represents H141A, E represents H211A, and F represents H276A.


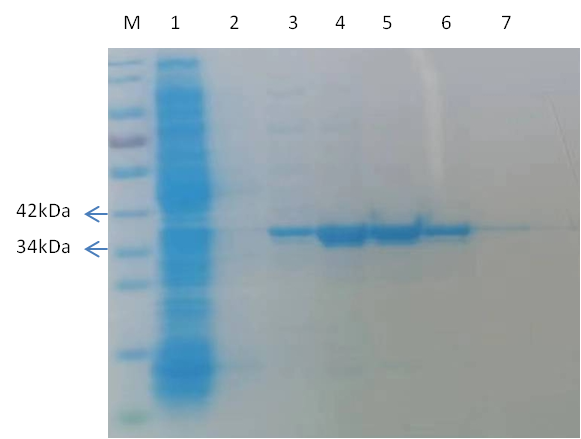


**Figure S2 Analysis of the size and purification of recombinant CAR-2 protein** M indicates Protein marker ; 1, 2, 3, 4, 5, 6, 7 indicate that the elution concentrations of imidazole are 20 mM, 50 mM, 100 mM, 200 mM, 200 mM, 300 mM, and 400 mM, respectively.


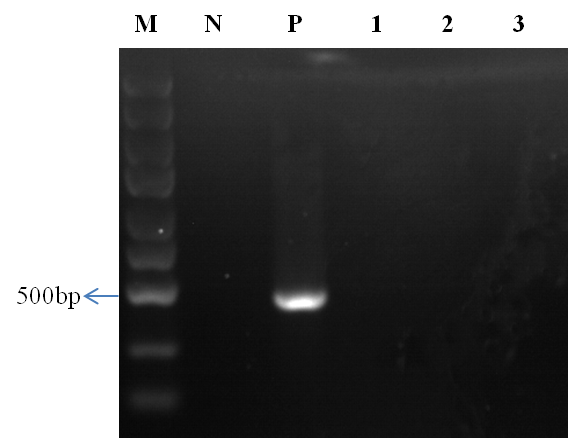


**Figure S3 PCR Validation of *CARR* Gene Knockout in *P. diazotrophicus*** M indicates DNA marker，N indicates the negative control, P indicates the positive control, and 1,2,3 indicate the test strains 1,2,3 respectively.


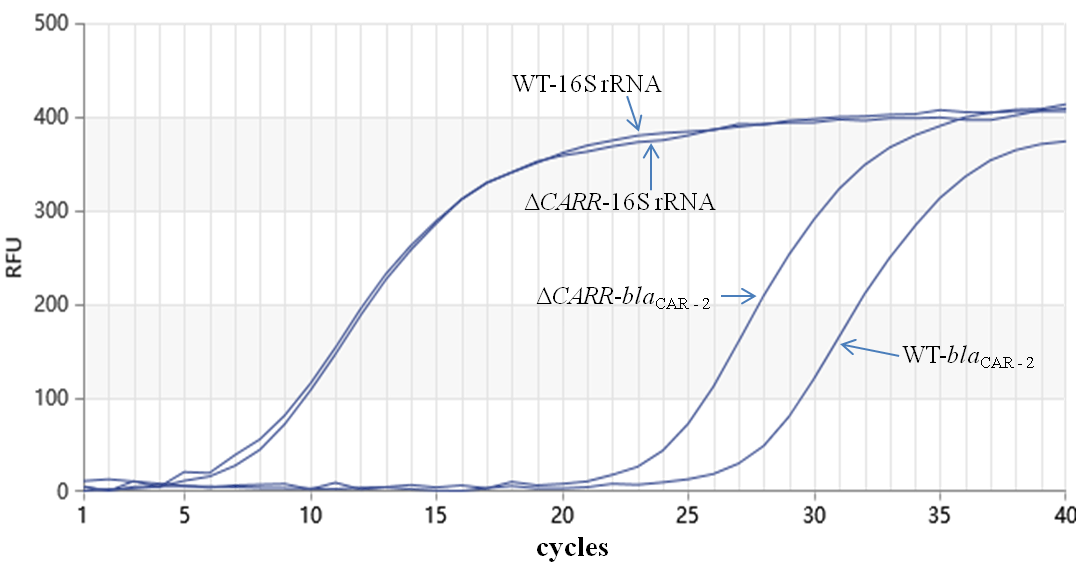


**Figure S4 Real - time amplification curves of 16S rRNA and *bla*_CAR-2_ of *P. diazotrophicus* wild strain and Δ*CARR* strain** WT indicates wild strain and Δ*CARR* indicates Δ*CARR* strain
